# Supplementary material for: The Biological Context of C-Reactive Protein as a Prognostic Marker in Renal Cell Carcinoma: Studies on the Acute Phase Cytokine Profile
Source: Cancers (Basel). 2020 Jul 19;12(7):1961. doi: 10.3390/cancers12071961 (PMC7409073; doi:10.3390/cancers12071961)
Supplement: Supplementary file 1 [file cancers-12-01961-s001.pdf]

The Biological Context of C-Reactive Protein as a Prognostic Marker in Renal Cell Carcinoma: Studies of the Acute Phase Cytokine Profile

Table S1. Attrition analysis—a comparison of preoperative clinical and biological characteristics for all renal cancer patients with and without available preoperative serum samples during the defined time period. The results are presented as mean ± standard error of the mean (continuous variables) or as the percentage of the patients.

PARAMETER	Available serum sample (*n* = 118)	No available sample (*n* = 36)	*p*-Value
Age at diagnosis-surgery (years)	62.7 ±1.3	59.6 ±2.4	0.252
Male gender	81%	67%	0.093
BMI at operation (kg/height in m^2)	27.2 ±0.4	25.9 ±0.6	0.145
Primary tumor size (cm)	6.3 ±0.3	6.2 ±0.6	0.804
Type of surgery			0.321
Radical nephrectomy	68%	58%	
Partial nephrectomy	32%	42%	
ASA score			0.581
1 and 2	81%	81%	
3 and 4	19%	19%	
Charlson comorbidity index			0.284
0-1	65%	67%	
≥2	35%	33%	
Stage			0.438
I-II	78%	78%	
III-IV	22%	22%	
Fuhrman nuclear grading			
1-2	53%	53%	1.000
3-4	47%	47%	
Histological necrosis			0.430
No necrosis	66%	58%	
Necrosis present	34%	42%	
Serum CRP level (mg/L)	11.7 ±2.5*	12.7 ±5.5**	0.852

* *n* = 117
** *n* = 33

Table S2. Classification of the 118 patients with renal cancer—a presentation of staging. The 97 patients included in our present cytokine studies were randomly selected from this population-based cohort. The table summarizes the tumor stadium and the TNM (tumor, nodal, metastasis) stage. The results are presented as the number of patients with the percentage in parenthesis, we present the results for all patients and the patients without metastases (see also Table 1; for additional details about the classification see 7th edition from 2010 in [36,37]).

	**All patients (*n* = 118)**	**Patients without metastases (*n* = 109)**
**Staging of the renal tumor**		
T1a (tumor only in the kidney, diameter ≤ 4 cm at its largest area)	54 (45.8)	54 (49.5)
T1b (tumor only in the kidney, diameter > 4 but ≤ 7 cm)	27 (22.9)	25 (22.9)
T2a (tumor only in the kidney, greatest dimension > 7 but ≤ 10 cm)	15 (12.7)	13 (11.9)
T2b (tumor only in the kidney, > 10 cm)	4 (3.4)	4 (3.7)
T3a (growth outside the kidney but not beyond Gerota's fascia)	13 (11.0)	10 (9.2)
T3b (growth into the renal vein below the diaphagm)	1 (0.8)	-
T4 (tumor invades beyond Gerota's fascia, extension into the adrenal gland)	4 (3.4)	3 (2.8)
**TNM stage**		
I (tumor stage T1, no involvement of lymph nodes, no metastases)	79 (66.9)	79 (72.5)
II (tumor stage T2, no involvement of lymph nodes, no metastases)	17 (14.4)	17 (15.6)
III (Either tumor stage T3 with no involvement of lymph nodes and no metastases; or tumor stage T1-T3, but with lymph node involvement and no metastases)	10 (8.5)	10 (9.2)
IV (Either tumor stage T4 independent of the nodal status but no metastases; or metastases independent of the tumor and nodal status)	12 (10.2)	3 (2.8)

Table S3. Serum CRP levels for patients with renal cell carcinoma; a summary of CRP correlation analyses.

**Parameter**	**Kendall's τ**	***p*-Value**
Age, *n* = 116	-0.018	0.788
ECOG classification, *n* = 116	**0.283**	<0.0005
ASA score, *n* = 116	0.215	0.006
Charlson comorbidity index, *n* = 116	-0.033	0.652
Fuhrman grade, *n* = 116	0.155	0.041
**Tumor stage, *n* = 116**	**0.315**	**<0.0005**
N stage, *n* = 116	-0.144	0.072
**Histological necrosis, *n* = 116**	**0.332**	**<0.0005**
IL33Rα, *n* = 94	0.173	0.019
IL1RA, *n* = 95	0.246	0.001
TNFα, *n* = 95	-0.027	0.716
**IL6, *n* = 116**	**0.301**	**<0.0005**
IL6Rα, *n* = 95	-0.039	0.596
gp130, *n* = 95	-0.041	0.573
IL27, *n* = 95	0.087	0.240
IL31, *n* = 95	0.008	0.911
OSM, *n* = 95	-0.034	0.649
CNTF, *n* = 95	-0.120	0.121

Table S4. Unsupervised hierarchical cluster analysis based on the preoperative serum levels of IL6 family mediators—a comparison of two main subsets of renal cancer patients referred to as CNTF^{high}IL6^{high} and CNTF^{low}IL6^{low}, respectively. The cluster analysis identified two main patient clusters, and each of these clusters could be further divided into a sub-cluster with low IL6/CNTF levels or relatively high levels of these two cytokines. Based on these results we classified the patients into two main subsets referred to as CNTF^{high}IL6^{high} and CNTF^{low}IL6^{low}, respectively. The table compares the cytokine levels for these two patient subsets. The results are presented as the median and range for each of the mediators together with the corresponding p-value (Mann-Whitney U test).

	**CNTF^{high}IL6^{high} (*n* = 42)**	**CNTF^{low}IL6^{low} (*n* = 55)**	***p*-Value**
**gp130**	91,032 (33,865-121,962)	91,380 (22,606-113,815)	0.754
**IL6Rα**	33,695 (22,510-48,588)	34,602 (17,789-46,610)	0.398
**IL6**	3.8 (0.4-16.3)	3.3 (0.0-73.2)	0.922
**IL27**	652 (254-1040)	710 (365-2738)	0.077
**IL31**	208 (83-584)	183 (87-410)	0.613
**OSM**	5817 (4500-7911)	5769 (3827-7023)	0.657
**CNTF**	490 (98-1281)	199 (98-2555)	0.178
**CRP, *n* = 41 / 54**	3 (1-48)	4 (1-220)	0.186
**IL33Rα, *n* = 42 / 54**	23,027 (7053-75,572)	23,156 (7731-162,569)	0.924
**IL1RA**	656 (281-1493)	736 (371-2711)	**0.027**
**TNFα**	24.2 (6.8-37.2)	26.2 (13.5-37.9)	**0.047**

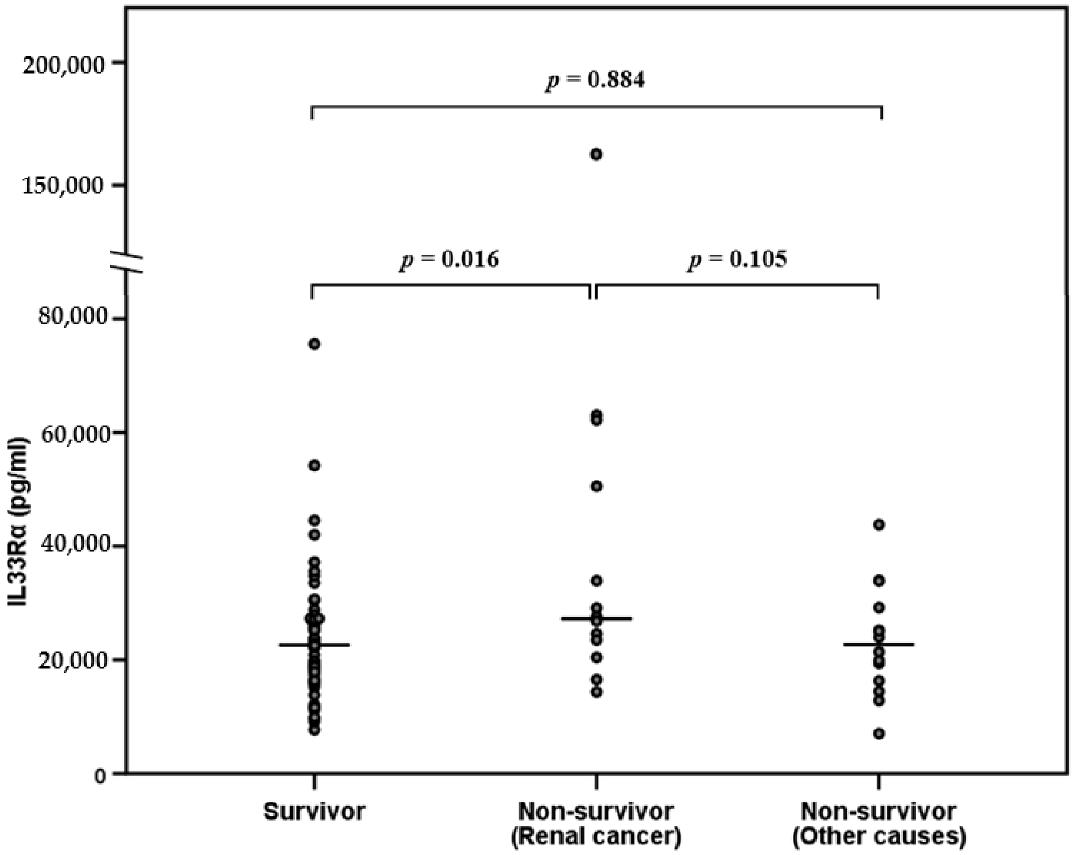

Figure S1. Serum levels of IL33Rα for patients with renal cell carcinoma; a comparison of survivors and non-survivors. The figure compares the levels for patients with cancer-free long-term survival (left); patients dying from their malignant disease (non-survivors, middle figure), i.e. patients with metastatic disease at the time of diagnosis and patients later dying for cancer progression/relapse; patients dying from other causes during the observation period (right, non-survivors other causes). The p-values from the statistical comparisons are indicated in the figure.
